# Complete Draft Genome Sequence of *Escherichia coli* KRX, a Strain for Efficient Cloning and High-Yield Expression of Proteins under Control of the T7 RNA Polymerase

**DOI:** 10.1128/genomeA.00933-17

**Published:** 2017-09-28

**Authors:** Jan-Philipp Schwarzhans, Daniel Wibberg, Anika Winkler, Jörn Kalinowski, Karl Friehs

**Affiliations:** a Fermentation Engineering, Bielefeld University, Bielefeld, Germany; b Microbial Genomics and Biotechnology, Center for Biotechnology, Bielefeld University, Bielefeld, Germany; c Genome Research of Industrial Microorganisms, Center for Biotechnology, Bielefeld University, Bielefeld, Germany; d Center for Biotechnology (CeBiTec), Bielefeld University, Bielefeld, Germany

## Abstract

*Escherichia coli* KRX is a strain offering both a high transformation efficiency and the possibility to produce the target protein to high yields in one host, avoiding additional cloning steps. Here, the draft genome sequence of *E. coli* KRX is presented and provides the genetic basis for additional biotechnological applications.

## GENOME ANNOUNCEMENT

*Escherichia coli* KRX is a commercially available derivate of *E. coli* K-12, created by Promega (catalog no. L3002 [Madison, WI, USA]). It combines characteristics that make it a suitable host for efficient plasmid transformation and propagation, as well as high-yield protein production with tight regulation. KRX is widely used for protein expression and protein evolution experiments, e.g., for cytochrome P450, peptide synthases, laccases, and Argonaute proteins (1–4), in certain cases showing markedly higher yields than other *E. coli* strains (5).

KRX enables high transformation efficiencies via the popular *recA1*, *endA1*, *hsdr17*, and *e14*^−^ gene alleles, preventing the degradation of plasmid DNA and unwanted recombination events (6).

Using the T7 RNA polymerase under control of the rhamnose-inducible promoter (*rha*P_BAD_), high protein yields can be achieved. *rha*P_BAD_ is activated by rhamnose and subjected to catabolite repression by glucose, enabling the use of autoinduction media. In *E. coli* KRX, the genes involved in the rhamnose metabolism have been deleted, ensuring a long induction phase.

While the genome of *E. coli* K-12 MG1655 is already publicly available (7), the genome of *E. coli* KRX shows some concise differences. Therefore, in this study, the draft genome sequence of *E. coli* KRX was established on the Illumina MiSeq system. A paired-end MiSeq sequencing run (2 × 300 bases) yielded 757,408 reads, accounting for 227.22 Mb of total sequence information. Assembly of all sequence reads applying the GS *De Novo* Assembler software (version 2.8) and reference mapping using r2cat (8) resulted in the draft sequences of the chromosome and the F plasmid of *E. coli* KRX, comprising 68 scaffolds and 110 contigs. Annotation of the chromosome was accomplished within the GenDB platform (9). The total size for the chromosome was determined to be 4,531,656 bp, featuring a G+C content of 50.78%. Gene prediction revealed 4,232 coding sequences (CDSs), 79 tRNA genes, and 5 rRNA genes. The F plasmid draft sequence has a size of 94,304 bp, features a G+C content of 48.01%, and contains 97 predicted protein-coding genes.

Analysis and interpretation of the *E. coli* KRX genome sequence within GenDB resulted in the identification of most described changes in comparison to *E. coli* K-12 MG1655. In contrast to the published genotype ([F′ *traD36* Δ*ompP proA*^*+*^* B*^*+*^* lacIq* Δ(*lacZ*)M15] Δ*ompT endA1 recA1 gyrA96* (*nal*^*r*^) *thi-1 hsdR17* (r_K_^−^ m_K_^*+*^) *e14*^−^ (*mcrA*^*–*^) *relA1 supE44* Δ(*lac-proAB*) Δ(*rha*_BAD_)::T7 RNA polymerase), no mutations in the *hsdR* and *relA* genes were detected. Interestingly, *E. coli* DH1 (GenBank accession no. CP001637), a strain that should harbor the same *hsdR17* and *relA1* alleles as KRX, also displays the native forms of these genes. This indicates that in a step leading to the creation of *E. coli* DH1 and *E. coli* KRX, these mutations were lost or incorrectly attributed. *E. coli* KRX contains a different mutation (Leu232Pro) instead of the one described in the literature for the *recA1* allele (Gly160Asp) (6). The genes *proA* and *proB* were located on the chromosome rather than on the F plasmid, potentially as a result of a reinsertion of these genes via homologous recombination.

### Accession number(s).

The *E. coli* KRX draft genome sequence was deposited in the EMBL database under the accession numbers CYPX01000001 to CYPX01000103.
